# Cardiac Findings in Acute Yellow Oleander Poisoning

**DOI:** 10.4103/0975-3583.59982

**Published:** 2010

**Authors:** Jalal Zamani, Amir Aslani

**Affiliations:** * Cardiology Department, Shiraz University of Medical Sciences, Shiraz, Iran

**Keywords:** Yellow Oleander, Digoxin, Poisoning

## Abstract

**Background:—:**

The Yellow Oleander is an ornamental tree that is common throughout the tropics. Ingestion of its seeds results in a clinical picture similar to digoxin toxicity.

**Objectives:—:**

The aim of this study was to evaluate cardiac findings in acute Yellow Oleander poisoning.

**Methods and Materials: —:**

A total of 21 patients with history of Yellow Oleander ingestion were enrolled in this study.

**Results:—:**

All symptomatic patients had conduction defects affecting the sinus node, theatrio-ventricular node or both. Patients showing cardiac arrhythmias that required specific management had significantly higher serum potassium concentrations.

**Conclusion: —:**

Most of the symptomatic patients had conduction defects affecting sinus or atrio-ventricular nodes but few had atrial or ventricular arrhythmias typical of digoxin poisoning.

## INTRODUCTION

The Yellow Oleander is an ornamental tree of the Apocyanaceae family that is common throughout the tropics.^1^ It contains cardiac glycosides that are toxic to cardiac myocytes and autonomic nervous system.^1^,^2^ Ingestion of its seeds results in poisoning similar to digoxin toxicity.^1^,^3^ Severely affected patients may manifest as resistant ventricular fibrillation.^1^,^4^ Intermediate poisoning may manifest as first degree atrio-ventricular (AV) block with progression to AV dissociation.^3^,^5^ The aim of this study was to evaluate cardiac findings and management of acute Yellow Oleander poisoning in southern Iran.

## MATERIAL AND METHODS

### Study Population

A total of 21 patients [53 ± 9.7 yrs; 17 female] were seen on admission to the Faghihi General Hospital with facilities for temporary pacing insertion from 1994 to 1998.

### Electrocardiographic Monitoring

At this hospital, 12-lead standard electrocardiography (INNOMED Medical ECG machine) and 2-lead ECG monitoring were taken during the standard work up of each patient.

### Blood Samples

Blood samples were collected during the standard work up of each patient. Five milliliters (5ml) venous blood was collected at 9:00AM every morning after overnight fast. Serums were isolated by centrifuging in a laboratory centrifuge at 2000g for three (3) minutes after blood clotting and retraction at room temperature. Serum potassium (K^+^), Sodium (Na^+^) and Calcium (Ca^2+^) were analyzed at the Department of Chemical Laboratory [Faghihi Hospital]. Serum bicarbonate (HCO3–) was determined by arterial blood gas measurement. Renal function including blood urea nitrogen [BUN], creatinine and liver function indices including SGOT, SGPT, PT and serum protein were analyzed in the clinical laboratory of Faghihi General Hospital using standard automated techniques.

### Statistical Analysis

Differences between the two groups (patients with significant arrhythmia vs. patients with insignificant arrhythmia) were analyzed with pair-wise comparisons. Baseline results are presented as counts and percentages and as mean ± SD for continuous variables. A P value < 0.05 was considered significant.

## RESULTS

A total of 21 patients [53 ± 9.7 yrs; 17 female] with a history of Yellow Oleander seed ingestion presenting to Faghihi General Hospital were enrolled in the study. A normal ECG was found on presentation in 39% of patients. In the remaining 61%, conduction abnormalities of the sinus node or AV node were detected [Table-1]. All patients with second or third degree AV block, prominent sinus bradycardia [< 40 /min], atrial or ventricular tachyarrhythmias were transferred to coronary care unit. Mean serum potassium concentration was significantly higher in patients with significant cardiac arrhythmias that required specific management [transfer to CCU, temporary pacemaker insertion and defibrillation] [Table-2]. Mean serum sodium and magnesium concentrations were not different between the two groups [Table-2].

**Table-1:** Cardiac arrhythmias in 21 patients with acute Yellow Oleander poisoning

Abnormal Rhythm	*n(%)*
Sinus Bradycardia	9 (42)
Sinus Arrest / Exit Block	5 (23)
Atrial Fibrillation	3 (14)
Junctional Rhythm	11 (52)
First-degree AV Block	9 (42)
Second-degree AV Block	6 (28)
Third-degree AV Block	4 (19)
Ventricular Ectopy	18 (85)
Ventricular Tachycardia	3 (14)
Ventricular Fibrillation	1 (4)
	

**Table-2:** Concentration of Serum Biomarkers

	Patients with Significan Cardiac Arrhythmia	Patients with Insignificant Cardiac Arrhythmia	*P*
Potassium	5.7 ± 0.9	4.1 ± 0.7	0.03
Sodium	136.7 ± 9.5	134.7 ± 8.9	NS
Magnesium	2.4 ± 0.5	2.1 ± 0.7	NS
Bicarbonate	21.2 ± 2.6	22.8 ± 3.4	NS
BUN	13.7 ± 3.4	15.7 ± 4.1	NS
Creatinine	1.4 ± 0.3	1.4 ± 0.5	NS
SGPT	22.7 ± 11.3	26.7 ± 8.8	NS
PT	12.3 ± 1.4	13.1 ± 1.2	NS

*Data are presented as mean* ± *SD; NS = not significant; BUN=blood urea nitrogen; PT=partial thrombin time*

## DISCUSSION

In nature, a wide variety of cardio-tonic steroids is found in plants, the insects that feed on them and in the parotid glands and skin of some toads.^2^,^6^ All these natural drugs contain a steroid nucleus with a lactone ring, five-membered in the case of cardenolides, six-membered in bufadienolides.^2^,^7^,^8^ The cardiac glycosides have a carbohydrate or sugar moiety attached through an oxygen bridge to carbon 3 of the ‘A’ ring of the steroid.^2^,^4^,^9^,^10^ The myocardial effects of these compounds are attributable to increased intracellular concentrations of Ca^2+^ and Na^+^ resulting from inhibition of the trans-membrane Na^+^ /K^+^ ATPase pump(2,10,11,12). The digitalis glycosides are by far the best known of the cardiac glycosides but many hundreds of others have been identified in different species of plants from at least 12 different families.^2^,^13^ The Apo-cyanaceae is source of African arrow poisons and also contains many of the most beautiful but deadly tropical flowers such as Yellow Oleander.^2^,^13^–^16^. The Yellow Oleander contains at least eight different cardiac glycosides.^2^,^10^ All parts of the plant are dangerous, especially the seeds. Ingestion of Oleander seeds or leaves is a common cause of accidental poisoning worldwide, particularly among children.^2^,^17^ The Oleander has been used for suicide, homicide, abortion and as herbal remedies in India, Thailand, Brazil and elsewhere.^2^,^11^,^15^ Yellow Oleander glycosides proved effective in patients with heart failure and atrial fibrillation. However, digitoxin or ouabains have been preferred because of less frequent gastrointestinal side-effects.

Most of our patients were healthy before their poisoning. This differs markedly from patients with digoxin poisoning and majority of patients with digoxin overdose are on multiple drug treatment. As a result of the patients’ young age and previously healthy state, the cardiac arrhythmias induced by oleander poisoning are unlikely to result from pre-existing conditions. Most symptomatic patients had conduction defects affecting the sinus or AV node and in 27% of the severe cases, both nodes were significantly affected.

Rhythms characteristic of Mobitz type II AV conduction block, although reported to be rare in isolated digoxin poisoning, occurred in several cases of the oleander poisoning.^1^,^7^
